# Effects of Intravenous Thiopentone Versus Etomidate on Modified Electroconvulsive Therapy (MECT) in Patients With Major Depressive Disorder

**DOI:** 10.7759/cureus.96428

**Published:** 2025-11-09

**Authors:** Balapriya Boopathy, Suneeth P Lazarus, Dilip Chandar, Balasubramanian Shanmugam

**Affiliations:** 1 Anesthesiology, Sri Manakula Vinayagar Medical College and Hospital, Puducherry, IND

**Keywords:** depression, electroconvulsive therapy, etomidate, inducible seizure, thiopentone

## Abstract

Background and aim

Electroconvulsive therapy (ECT) has a well-established role in the management of patients who do not respond to psychopharmacological treatment. The therapeutic effect of ECT on major depressive disorder is widely investigated; the search for an ideal induction agent for ECT is an ongoing process. The present study was conducted to compare the effects of 3 mg/kg of intravenous thiopentone versus 0.2 mg/kg of intravenous etomidate on modified electroconvulsive therapy (MECT) in patients with major depressive disorder.

Methods

The present study was carried out as a double-blinded randomized study on 40 patients diagnosed with major depressive disorder. The study was approved by the institutional ethics committee, and written informed consent from each patient was obtained before the study. A total of 40 patients were divided into two groups of 20 each by block randomization. Group A patients (n = 20) received IV etomidate 0.2 mg/kg. Group B (n = 20) patients received IV thiopentone 3 mg/kg. After induction and muscle relaxation, electrical stimulus was applied, and the duration of seizure quality was measured using the cuff method.

Results

All statistical tests were done with IBM SPSS Statistics for Windows, Version 24 (Released 2016; IBM Corp., Armonk, New York, United States). The average seizure duration was 54 seconds in group A (etomidate), and it was 42 seconds in group B (thiopentone). Etomidate showed longer seizure duration compared to thiopentone with a significant p-value of 0.001 using an independent-samples T-test.

Conclusion

Etomidate, as an induction agent for patients with major depressive disorder, showed good seizure quality compared to thiopentone.

## Introduction

Electroconvulsive therapy (ECT) is the treatment of choice for drug-resistant depression, schizophrenia, and mania [1]. Initially, “unmodified ECT” was practiced without muscle relaxation, and the patients were kept conscious during the procedure. Physical and psychological trauma, such as muscle rigidity, bone fractures, hypoxia, cardiac arrhythmias, and vertebral compression of the mid thorax, were observed during ECT. To increase patient acceptability and safety in the 1950s and 1960s, refinements, including anesthetic muscle relaxants and medications, were introduced [2]. An Ideal induction agent for ECT should have a rapid onset and offset, with minimal hemodynamic changes without affecting the seizure duration or its threshold [2]. There are multiple intravenous anesthetic agents to provide anesthesia during ECT, with a few agents having a pitfall of reducing the seizure duration. In this study, we made an attempt to identify a suitable anesthetic agent for ECT by comparing etomidate and thiopentone. The primary objective of the study was to compare the seizure duration of 2.5%, 3 mg/kg of intravenous thiopentone, and 0.2 mg/kg of intravenous etomidate on modified ECT (MECT) in patients with major depressive disorder, and the secondary objective was to evaluate the safety profile by assessing the hemodynamic variables, electrical charge applied, post-anesthetic recovery, post-ictal confusion and adverse reactions. 

## Materials and methods

The present prospective randomized double-blinded study was conducted in a tertiary teaching hospital in South India after obtaining approval from the hospital’s ethical and research committees: EC/107/2018. The study was registered under the Clinical Trials Registry of India with registration number CTRI/2019/04/018687. A total of 40 American Society of Anaesthesiologists physical status I and II patients aged between 18 and 55 years, with major depressive disorder, undergoing MECT were enrolled in the study. Computer randomization with allocation concealment was carried out, and all patients were evenly assigned into two groups of equal size. Patients were informed about the nature of the study, and an informed, valid written consent was obtained either from the patient with proper cognition or from the patient’s caregiver in patients who were in a state of inability to give proper consent. Patients with known allergy to sulpha drugs, with a history of porphyria, and with a history of asthma, pregnant/lactating women, and those who are reluctant to participate were excluded from the study. On arrival of the patient in the operating room, all standard monitors were attached, baseline heart rate (HR), mean arterial pressure, and oxygen saturation (SpO2) were recorded. Anesthesia was administered by an anesthesiologist who was not involved in the study. He/she had prepared the study solution according to the code received by the patient. The patients were premedicated with an injection of glycopyrrolate 0.2 mg intravenously. After preoxygenation, the study drug was administered by the attending anesthesiologist. A pneumatic tourniquet was placed on the right leg and was inflated to twice the systolic blood pressure of the patient to isolate the right leg before administration of a neuromuscular blocking agent to assist in monitoring the peripheral seizure. Neuromuscular relaxation was achieved by intravenous succinylcholine 0.5-1 mg/kg. When relaxation was adequate, with the use of Bain’s circuit, satisfactory mask ventilation with oxygen was ensured, a bite block was placed, and a stimulus was delivered to induce the seizure. Two psychiatrists were involved in the study; one psychiatrist administered the ECT by placing electrodes over the bifrontal area. The second psychiatrist, who was unaware of the study drug, entered the theater after induction and monitored the peripheral seizure by observing the patient. During the session, hemodynamic parameters, duration of seizure, and electrical charge applied were also noted. After the procedure, ventilation with oxygen by mask was continued until the patient awakened and was breathing adequately. The patients were monitored in a fully staffed and well-equipped recovery room until the specific discharge criteria were met. The principal investigator later collected all the intraoperative and postoperative data after the patient was shifted to the ward from the recovery area. Even in successive sessions, the same mode of anesthesia with the study drug was administered, and the outcomes were observed.

Statistical analysis

**Figure 1:**
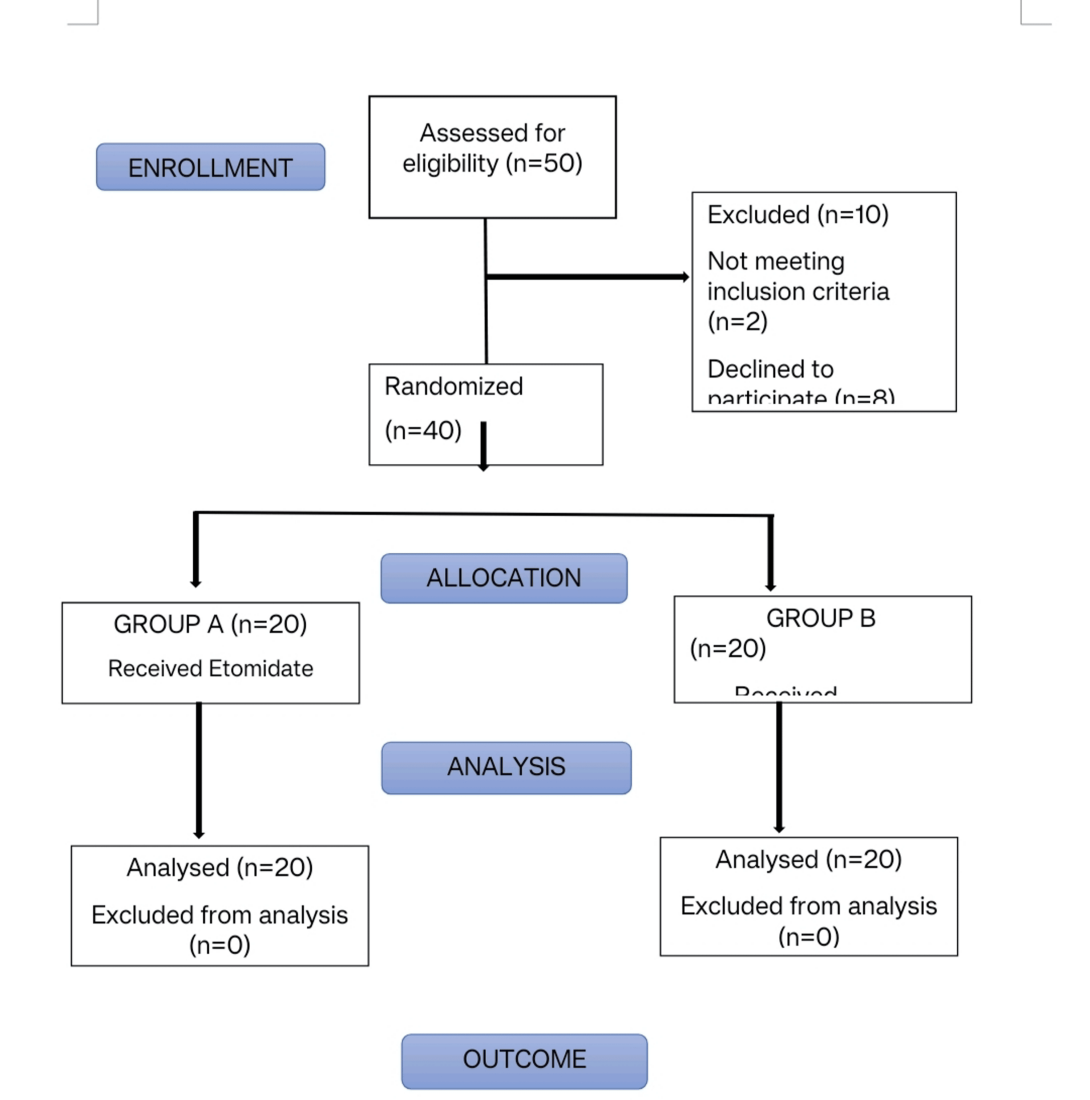
Consort flow chart for the study

The sample size was calculated using OpenEPI Software version 3.0, considering the impact of thiopentone and etomidate on seizure duration, which was 26.69 (+/- 9.7) seconds and 35.92 (+/- 9.2) seconds, respectively, using the cuff method (secs) as per a previous study [3]. With a 95% confidence interval and 80% power, considering the 10% of nonresponsiveness, the sample size was calculated to be 40 as the final figure, and 20 patients were included in each group. At the end of the study, all the data were entered in MS Excel (Microsoft Corporation, Redmond, Washington, United States) and analyzed using statistical software Epi Info 3.0 and IBM SPSS Statistics for Windows, Version 24 (Released 2016; IBM Corp., Armonk, New York, United States). The study data were analyzed statistically by using the chi-square test, independent-samples T-test, and Fisher’s exact test. Data was expressed as mean +/-SD. A p-value of <0.05 was considered significant. All the patients after screening were enrolled in the study (Figure 1).

## Results

Patients from both groups had a comparable demographic profile with no significance statistically. All the patients who were enrolled completed the study without any dropouts. Regarding hemodynamic parameters, the etomidate group showed stable diastolic blood pressure as compared to the thiopentone group. However, the electrical stimulus required and seizure duration among the groups showed a significant difference. The results are summarized in tables and graphs.

Table 1 shows the total electrical charges applied to both groups to induce seizure activity. The electrical charge to induce or initiate a seizure activity was quantitatively less in group A compared to that in group B, which was statistically significant (p = 0.048).

**Table 1:** Electrical stimulus in terms of volts applied among the groups p-value based on Fisher’s exact test

Electrical stimulus in volts	Group A, n (%)	Group B, n (%)	p-value
6V	11 (55%)	4 (20%)	0.04837
8V	9 (45%)	15 (75%)	
12V	0	1 (5%)	
Electrical stimulus in volts	Group A n (%)	Group B n (%)	p value
6V	11 (55%)	4 (20%)	0.04837
8V	9 (45%)	15 (75%)	
12V	0	1 (5%)	

Tables 2-3 show the statistical analysis with respect to mean seizure duration among the groups. Group A had a mean duration of 54.55 + 5.246 seconds, and group B had a mean duration of 42.35 + 9.343 seconds. Seizure duration was significantly longer in group A than in group B, which was statistically significant (p-value = 0.005).

**Table 2:** Mean seizure duration among groups p-value based on an independent-samples T-test

	Mean (seconds)	Standard deviation (seconds)	Standard error mean (seconds)	p-value
Group A	54.550	5.246	1.173	0.005
Group B	42.350	9.343	2.089	
T-test	4.688			

**Table 3:** : Seizure duration among the groups p-value based on Fisher’s exact test

Duration (in secs)	Group A, n (%)	Group B, n (%)	p-value
<39 secs	0	11 (55%)	0.001
40-49 secs	2 (10%)	3 (5%)	
50-59 secs	4 (20%)	5 (25%)	
>60 secs	14 (70%)	1 (5%)	
Total	n = 20	n = 20	
Mean seizure duration	54 seconds	42 seconds	

**Figure 2:**
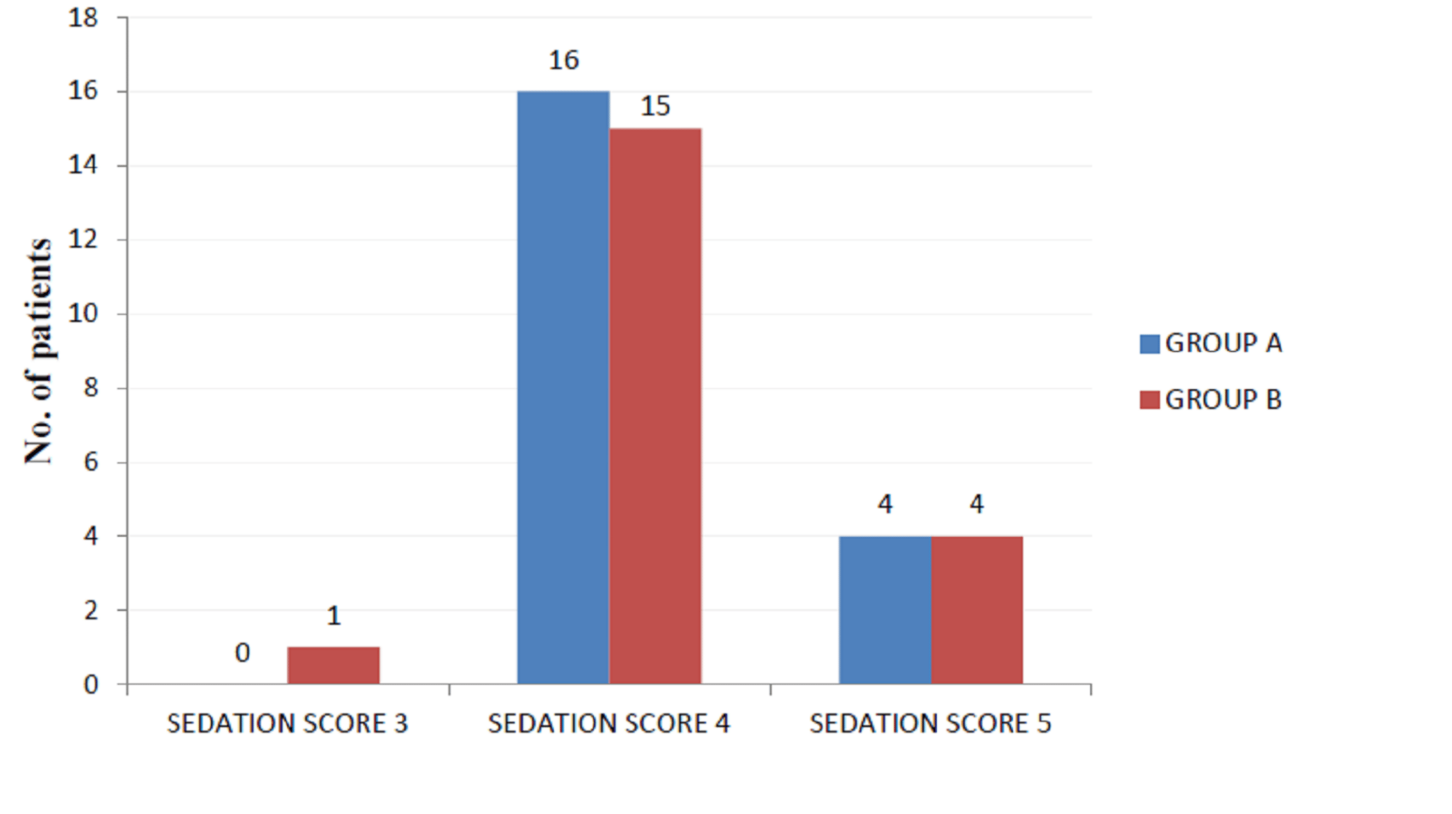
Sedation score

Sedation was assessed at the same intervals of time according to the observer’s assessment of alertness/sedation (OAA/S) scale (Figure 2).

With regard to post-ictal confusion among the groups, in group A, 16 patients developed post-ictal confusion, and only four patients developed post-ictal confusion in group B. No patients developed any obvious adverse reaction, both immediate and post-procedure follow-up. Table 4 shows changes in the mean arterial pressure in both groups. On comparison, there was a statistically significant rise in the mean arterial pressure in group B compared to group A.

**Table 4:** Mean arterial blood pressure distribution among the groups MAP: mean arterial pressure; SD: standard deviation p-value based on independent-samples T-test

Mean arterial blood pressure	Group A (n = 20) mean (+ SD)	Group B (n = 20) mean (+ SD)	p-value
Baseline MAP	86.30 (+9.02)	85.05 (+7.62)	0.639
2 mins MAP	98.45 (+5.47)	109.35 (+3.92)	0.001
5 mins MAP	97.60 (+4.35)	111.00 (+2.99)	0.001
10 mins MAP	97.45 (+3.47)	100.60 (+2.56)	0.002

Table 5 shows changes in heart rate in both groups. On comparison, there was a statistically significant rise in heart rate in group B compared to group A, p = 0.001.

**Table 5:** Heart rate variations among the groups HR: heart rate; SD: standard deviation p-value based on an independent-samples T-test

Heart rate per minute	Group A (n = 20) mean 9+ SD)	Group B (n = 20) mean (+ SD)	p-value
Baseline HR	73.05 (+7.95)	74.80 (+8.53)	0.506
2 min HR	62.65 (+4.45)	77.15 (+7.65)	0.001
5 min HR	61.95 (+3.25)	77.25 (+8.81)	0.001
10 min HR	67.00 (+4.42)	77.00 (+8.89)	0.001

**Figure 3:**
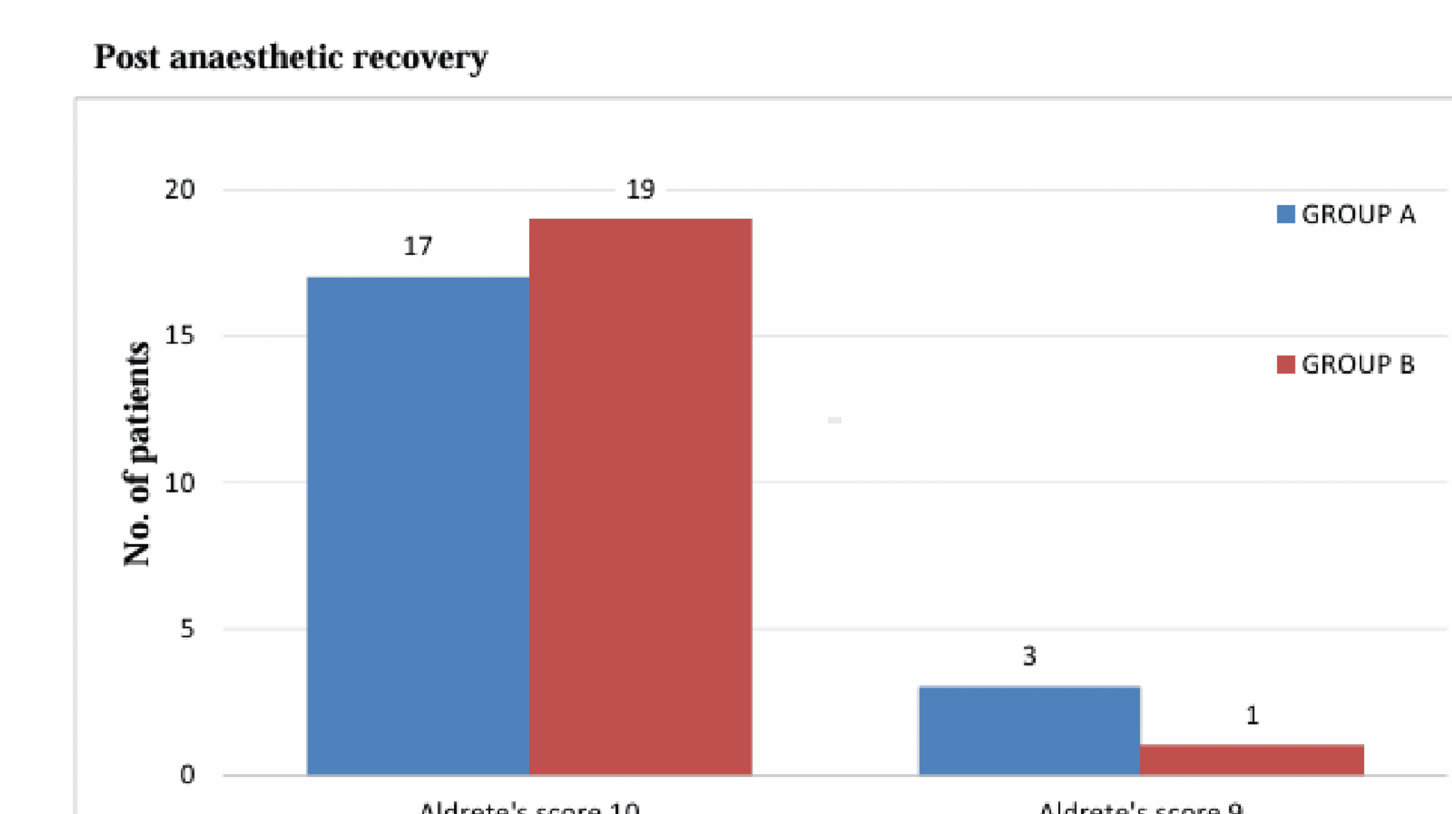
Aldrete score

Aldrete’s scoring system is a scoring method to assess the patient for discharge in the post-anesthesia care unit (PACU). Figure 3 shows the distribution of Aldrete scores between groups.

## Discussion

ECT is a well-established procedure for psychiatric patients who are not responding to conventional pharmacological treatment [3-5]. Though the benefits of ECT are proven, it has been less preferred in the recent past due to the nature of the procedure and the side effects associated with it [5-7]. Now ECT is re-emerging as a treatment of choice due to its modifications and is labelled as MECT. In MECT, the side effects associated with violent seizures are nullified by administering a short-acting muscle relaxant, and the stress associated with the ECT is reduced by co-administering a short-acting anesthetic agent.

The anesthesiologist's expertise is requisite for navigating the inherent procedural challenges and marked hemodynamic changes associated with MECT [7-11]. The therapeutic efficacy of MECT depends on the longevity of seizure activity. However, the controversy still exists whether the efficacy depends on duration or quality of seizure [12]. Patients during ECT initially exhibit parasympathetic discharges followed by sympathetic activation; the ideal induction agent should stabilize these sympathetic derangements and should maintain cardiovascular stability [12-15]. So the ideal anesthetic agent chosen for maintaining anesthesia during MECT should have a rapid onset and recovery with a short half-life and should be hemodynamically stable without affecting the seizure duration or its threshold.

The majority of induction agents have GABA agonistic activity, i.e., anticonvulsant activity, which, when used in MECT, results in a countertherapeutic effect. The search for the perfect induction agent for ECT has been an ongoing process. We wanted to evaluate etomidate as an induction agent for MECT for its seizure-mimetic effect and more cardiostable effect with rapid recovery. 

In our study, we observed that patients receiving etomidate as an induction agent had significantly longer seizure duration compared to the thiopentone group (p-value = <0.05). Abdollahi et al. [16], Conca et al. [17], and Saffer and Berk [18] reported similar findings to our study. Christensen et al [19] compared thiopental and etomidate in 10 patients with depression and reported longer seizure duration with etomidate. Trzepacz et al. [20] conducted a study on 28 depression patients and found that the mean seizure duration was significantly longer (p < 0.001) for etomidate compared with thiopental. We also observed that patients belonging to the etomidate group required significantly less electrical energy to induce a good seizure length activity compared to the thiopentone group (p < 0.05). 

In relation to hemodynamic changes, we found that the etomidate group showed a stable diastolic blood pressure and mean arterial blood pressure when compared to the thiopentone group. The only pitfall noted with the etomidate group is that it showed a prolonged recovery duration with post-ictal confusion. In the thiopentone group, there was a rapid recovery of consciousness with minimal confusion.

Etomidate usage as an induction agent for MECT may improve seizure duration in patients with major depressive disorder more than thiopentone [20-22]. However, there is a concern regarding the usage of etomidate as an induction agent is associated with adrenocortical suppression on prolonged infusions. In the present study, we used a single dosage of 0.2 mg/kg for induction only once to avoid that possible complication, although serial cortisol level monitoring would have provided more evidence for our observation. Past studies by Fragen et al. [23] and Wagner and White [24] reported that the adrenal suppression effect was reversible and lasted <6 hours in most cases. Wang et al. [25] checked serial cortisol levels during ECT and reported no significant reductions associated with the use of etomidate.

Strengths and limitations

Appropriate randomization procedures were followed to reduce the chance of selection bias. We used the cuff method to monitor the peripheral seizure activity; however, an EEG for central seizure activity would have been more appropriate. Further studies with a larger sample size and longer duration are required to investigate the long-term effects of these medications. Our study was limited to evaluating the seizure duration with two induction agents; we did not study the effect of the induction agent on depression outcome. Serial cortisol levels were not done to diagnose adrenocortical suppression because the dose of etomidate used in the study was comparatively low to incite adrenocortical suppression. 

## Conclusions

To conclude, as an induction agent for MECT, etomidate is associated with a longer duration of seizure requiring less electrical stimulation, which may be related to less adverse outcomes compared with thiopentone. In situations where etomidate and thiopentone are the choice of drugs for MECT, we propose that the use of etomidate might be favorable for better seizure outcome during MECT.
